# A novel directional-oriented method for predicting shear wave velocity through empirical rock physics relationship using geostatistics analysis

**DOI:** 10.1038/s41598-023-47016-9

**Published:** 2023-11-14

**Authors:** Esmael Makarian, Maryam Mirhashemi, Ayub Elyasi, Danial Mansourian, Reza Falahat, Ahmed E. Radwan, Ahmed El-Aal, Cunhui Fan, Hu Li

**Affiliations:** 1 Department of Mining Engineering, Sahand University of Technology, Tabriz, 94173-71946 Iran; 2 CAPE Consultant Group, Tehran, Iran; 3 Ph.D. Researcher in Mewbourne College of Earth and Energy, Oklahoma University, Norman, USA; 4 Faculty of Petroleum Engineering, Sahand University of Technology, Tabriz, Iran; 5 Faculty of Geography and Geology, Institute of Geological Sciences, Jagiellonian University, Gronostajowa 3a, 30-387 Kraków, Poland; 6 Civil Engineering Department, Faculty of Engineering, Najran University, P.O. Box: 1988, Najran, Saudi Arabia; 7 Science and Engineering Research Center, Najran University, Najran, Saudi Arabia; 8 School of Geoscience and Technology, Southwest Petroleum University, Chengdu, 610500 China

**Keywords:** Geophysics, Mathematics and computing

## Abstract

This study attempts to design a novel direction–oriented approach for estimating shear wave velocity (V_S_) through geostatistical methods (GM) using density employing geophysical log data. The research area involves three hydrocarbon wells drilled in carbonate reservoirs that are comprised of oil and water. Firstly, V_S_ was estimated using the four selected empirical rock physics relationships (ERR) in well A (target well), and then all results were evaluated by ten statistical benchmarks. All results show that the best ERR is Greenberg and Castagna, with R^2^ = 0.8104 and Correlation = 0.90, while Gardner's equation obtained the poorest results with R^2^ = 0.6766 and correlation = 0.82. Next, Gardner's method was improved through GM by employing Ordinary Kriging (OKr) in two directions in well A, and then Cross-Validation and Jack-knife methods (JKm and CVm, respectively) were used to assess OKr's performance and efficiency. Initially, CVm and JKm were employed to estimate Vs using the available density and its relationship with shear wave velocity, where the performance of CVm was better with R^2^ = 0.8865 and correlation = 0.94. In this step, some points from the original V_S_ were used to train the data. Finally, Vs was estimated through JKm and using the relationship between the shear wave velocity of two wells near the target well, including wells B and C; however, in this step, the original shear wave velocity of the target well was completely ignored. Reading the results, JKm could show excellent performance with R^2^ = 0.8503 and Corr = 0.922. In contrast to previous studies that used only Correlation and R-squared (R^2^), this study further provides accurate results by employing a wide range of statistical benchmarks to investigate all results. In contrast to traditional empirical rock physics relationships, the developed direction-oriented technique demonstrated improved predicted accuracy and robustness in the investigated carbonate field. This work demonstrates that GM can effectively estimate Vs and has a significant potential to enhance V_S_ estimation using density.

## Introduction

The subsurface studies, whether deep studies, such as hydrocarbon discovery, or surface surveys, such as sample water exploration and geotechnical issues, desperately need an accurate tool to investigate features, like determining underground structures or assessing rocks, fluids, and porous media properties^1,2^**.** P-wave (V_P_) and S-wave velocity (V_S_) are two important and versatile tools that are very efficient and useful in determining reservoir characteristics. To give more information, V_S_ is an essential parameter in determining subsurface structures in size and geometry^3^**.** In reservoir characterization, V_S_ helps determine lithofacies, fluid properties, and electrical resistivity using the Vs–Vp relation, which is regarded as a magic tool^4^**.** Moreover, the V_S_–porosity relationship can help assess the sorting and volume of cementation, especially for sandstone reservoirs^5,6^. The shear wave velocity is also widely used in reservoir geomechanics because it is considered a predominant factor in estimating the elastic modulus and building the geomechanical model of the reservoirs^7–9^.

On the other hand, the propagation of seismic wave velocity profoundly depends on the elastic properties of the porous medium, which is derived from the properties of both rocks and fluids; however, each seismic wave is affected by the factors according to its inherent characteristics^10,11^ An extensive diversity of direct and indirect methods is employed to calculate shear wave velocity. It can be measured directly in the laboratory by a core sample or by well–logging tools, not the least of which is the DSI (Dipole Sonic Imager)^12^. However, these methods have some restrictions and problems; for example, in some situations, such as horizontal and deviation wells, getting a core sample or performing well-logging operations would be difficult or impossible, and above all, they are time–consuming and expensive^13^**.**

Over the past few years, a wide range of indirect methods have been presented to tackle these problems. A myriad of empirical rock physical relations has been introduced to predict V_S,_ such as Borcher et al. (2005), Kerif (1990), Castagna et al. (1985), Pickett (1963), Greenberg and Castagna (1992), Gassmann (1951), Castagna and Backus (1993), and Han et al. (1989)^14–21^. These methods mainly employ P–wave velocity and lithology to predict V_S_ in different areas. Besides, thanks to the immense advance in science and technology, intelligent methods have been widely used to estimate V_S_, some of which are listed as follows: Taheri et al. (2022), Mehrad et al. (2022), Zhang and Ben‐Zion (2020), Wang and Cao (2021), Ebrahimi et al. (2022), Olayiwola and Sanuade (2021), Liu et al. (2021), Miah (2021), Azadpour et al. (2020), Yang et al. (2019), Anemangely et al. (2019)^22–32^. These methods usually utilize numerous inputs, for instance, gamma ray (GR), density (Rhob), V_P_ porosity, and resistivity, to get satisfactory and accurate results. Because they are input–based methods and work through discovering the relationship between input data and target parameters, studies show that the more input data, the better the results^22^. Geostatistical methods are other indirect methods that have mostly been used to estimate shear wave velocity profiles in surface and near-surface projects^33,34^. Maleki et al.^35^ generates a 3D shear wave velocity model for a well through the Kriging and Back Propagation Neural Network (BPNN) based on the correlation between P-wave velocity and V_S_. Most of the previous studies were conducted on only one well and used more than two sets of data to estimate the shear wave velocity. Scrutinizing previous studies reveals that not much attention was paid to estimating shear wave velocity through mere density because it does not have a good correlation with V_S_. Given those considerations, the main goal of this study is to discover a novel numerical direction to predict shear wave velocity using density as the least available data to save time and money through developing an empirical rock physics relationship (ERR) by geostatistical methods (G.M.). For this purpose, initially, S-wave velocity is estimated using a total of ERR provided by Gardner^36^, Castagna et al.^16^, Kerif^15^, and Greenberg and Castagna^18^ in the target well. Then, the poor-quality estimated data is improved through G.M., including the cross-validation method (CVm) and the Jack-knife method (JKm). In the third step, the shear wave velocity of the target well is estimated by the JKm using the densities of two nearby wells. In contrast to previous studies that used only correlation and R-squared (R^2^), this study further provides accurate results by employing a wide range of statistical benchmarks to investigate all results comprehensively and accurately. These benchmarks include mean absolute percentage error (MAPE), Margin of error (ME), mean absolute error (MAE), mean percentage error (MPE), the root mean squared error (RMSE), and Minimax. Besides, the mean and standard deviation (STDEV) for all estimations were considered. Because the new strategy in this article involves a lot of numerical methods, some statistical benchmarks are a normal and essential activity that must be done to evaluate results accurately.

### Geological information and data set description

**Figure 1:**
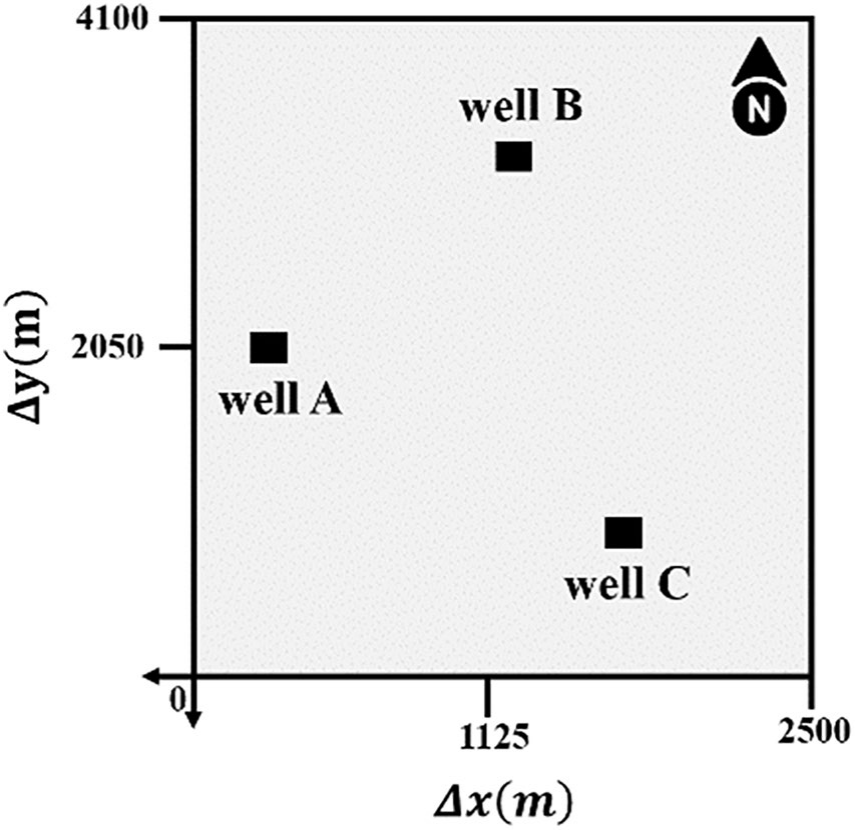
Location map of the used well in the study area and their relative distance.

The study was performed in the Persian Gulf's oil carbonate reservoir (here named R). Studies show numerous hydrocarbon reservoir formations exist mainly within carbonate sequences of different ages in the studied basin, ranging from the Triassic to the Tertiary^37^. The data set used in this study has been taken from the R reservoir, namely wells A, B, and C, which are close to each other (Fig. 1). Petrophysical analysis from core samples and well logging data show that roughly the selected wells in terms of lithology and fluids are the same. In all wells, limestone is responsible for the largest share of lithology with approximately 85%, followed by dolomite with almost 10%, and shale accounted for the smallest proportion of lithology (about 5%). All research wells mainly comprise water and oil, with a high oil–water ratio (OWR); however, negligible gas has been detected. Laboratory investigations based on thin section analysis proved that the pore type in the target formation is mainly interparticle and that there are no significant changes or fractures^38^. Tectonically, none of the tectonic processes, such as folding or faulting, have affected the studied area. This study uses well-logging data to predict V_S_ and selects well A as the target. In order to estimate S-wave velocity through empirical rock physics relationships (ERR) in the target well, P-wave velocity, density, and volume lithology information have been used, and for applying geostatistical methods (GM), density has been used.

## Methodology

S–wave velocity in subsurface projects may not be measured at all, or the measured value may have problems at some intervals or misses. In order to estimate S–wave velocity in this paper, two main approaches are employed: ERR and GM. In the former method, the S–wave velocity of well A was ignored and then estimated by some ERR based on different parameters, including density, P–wave velocity, and lithology, unlike the previous studies that only used V_P_ and lithology and did not consider density. The GM method improves the poorest results using the Ordinary Kriging method (OKr). Following this, CVm and JKm are obligated to evaluate the performance and efficiency of OKr. It must be mentioned that all results were evaluated and compared to the (measured) original V_S_ through ten different statistical benchmarks; however, in the previous studies, only R^2^ and Correlation were employed.

### Empirical rock physics relationship (ERR)

A seismic velocity such as a compressional (P) or shear (S) wave is mainly dependent on the characteristics of porous media, not the least of which are its elastic properties^39^. The velocity of the shear (S) wave can be defined by Eq. (1):

1 $$V_{S} = \sqrt {\frac{\mu }{\rho }}$$

where V_S_ is S-wave velocity, µ is shear modulus, and ρ is the density of a rock, these two sonic waves are further connected by concerning elastic properties:

2 $$\frac{{V_{P} }}{{V_{S} }} = \left( {\frac{K}{\mu } + \frac{4}{3}} \right)^{\frac{1}{2}}$$

3 $$\frac{{V_{P} }}{{V_{S} }} = \left( {\frac{1 - \vartheta }{{\frac{1}{2} - \vartheta }}} \right)^{\frac{1}{2}}$$

In Eqs. (2) and (3), K and µ are the bulk and shear modulus, and also ϑ is the Poisson ratio of the medium; the equations illustrate that the value of the velocity ratio is always more than one (V_P_/V_S_ > 1)^40^. The first section of this paper estimates S–wave velocity through the selected empirical relationship between V_P_ and density. It is worth mentioning that these equations were provided based on specific areas and conditions. This study obtained data from well–logging, including P–and S–wave velocities, densities, and volume logs consisting of calcite, dolomite, and shale. The employed rock physical equations are discussed below. Firstly, Kerif et al.^15^ provided an excellent relationship to estimate Vs as following Eq. (4) using the relation between V_P_ and V_S_:

4 $$V_{S} = \sqrt {a \times V_{P}^{2} - b}$$

where V_S_ is shear wave velocity (m/s), V_P_ is compressional wave velocity (m/s), and a and b are coefficients considered to have the following values: a = 0.331999 and b = 1743. One of the most important and widely used equations for estimating P–wave velocity is the Gardner (1947) relationship. Gardner^36^ generated an applied relationship between density and P–wave velocity based on Eq. (5):

5 $$\rho = \alpha \times V_{p}^{\beta }$$

where *ρ *is the bulk density (g/cm^3^), *V*_*p*_ is the sonic P-wave velocity (m/s), and *α* and $\beta$ are coefficients of 0.31 and 0.25, respectively (default values). Jaramillo^41^ tried to estimate P and S wave velocity by different empirical relationships using well-logging data in North American basins with various lithologies. For the carbonate section, he employed an equation according to Gardner's equation (1974) separately as Eq. (6) for estimating S wave velocities:

6 $$V_{S} = 10^{{\frac{\log \rho - \log a}{m}}}$$

In Eq. (6), V_S_ is shear wave velocity (m/s) $\rho$ is density (kg/m^3^), and a and m are localized coefficients, with 350 or divided constant and 0.25, respectively. He could not obtain favorable results to estimate V_S_ utilizing Eq. (6). The current research uses Eq. (6) to estimate V_S_ and improve its results using geostatistical methods.

Additionally, we have considered the equation given by Castagna et al. (1985) as Eq. (7):

7 $$V_{S} = a \times V_{P} + b$$

where we assumed the values of a and b are 0.86 and  − 1170, respectively.

Also, other equations are provided by Castagna et al. (1993) for carbonates as follows Eq. (8):

8 $$\begin{gathered} {\text{Limestone}}\quad V_{S} = 0.5508 \times V_{P}^{2} + 1.01677 \times V_{P} - 1.03049 \hfill \\ {\text{Dolomite}}\quad V_{S} = 0.58321 \times V_{P} - 0.07775 \hfill \\ \end{gathered}$$

Only V_P_ and density are involved in the equations as mentioned above^16^**.**

Lastly, Greenberg and Castagna's (G.C) fourth and final equations (1992) have provided a useful empirical relationship to estimate the shear wave velocity^18^. The equation, in addition to V_P_, has also been used in lithology and mineralogy based on Eq. (9):

9 $$Vs = \frac{1}{2}\left\{ {\left[ {\sum\limits_{i = 1}^{L} {X_{i} \sum\limits_{j = 0}^{{N_{i} }} {a_{ij} V_{p}^{j} } } } \right] + \left[ {\sum\limits_{i = 1}^{L} {X_{i} \left( {\sum\limits_{j = 0}^{{N_{i} }} {a_{ij} V_{p}^{j} } } \right)^{ - 1} } } \right]^{ - 1} } \right\}$$

In Eq. (9), L denotes the number of pure components in terms of lithology; X_i_ presents the volume proportion of lithological constituents; a_ij_ is an empirical regression coefficient relying upon lithology (Table 1); N_i_ refers to the order of polynomial for constituent i; and V_jP_ is the water-saturated P-wave velocity in the j rock facies. Finally, V_S_ is S-wave velocities (km/s) in composite brine-saturated, multimineral rock.

**Table 1:** The Greenberg–Castagna relations (1992) regression coefficients for V_S_ prediction.

Lithology	$a_{i2}$	$a_{i1}$	$a_{i0}$
Sandstone	0	0.80416	− 0.85588
Limestone	− 0.05508	1.01677	− 1.03049
Dolomite	0	0.58321	− 0.07775
Shale	0	0.76969	− 0.86735

In all the methods mentioned above, the relative volume of constituents and the type and mechanical features of different minerals were not included in estimations. However, these important factors are considered in Greenberg–Castagna's (1992) relationship.

### Geostatistics methods (GM)

Geostatistics is the statistical understanding of changes in time and space that mainly addresses spatial or temporal data sets and deals with spatially distributed and spatially correlated phenomena of the data^42^. Geostatistical methods (GM) initially strive to specify and quantify the spatial structure of valuable information, after which the target (needed) data are interpolated or predicted through near points considering their spatial structure^43^. Over the past few decades, GM has gained popularity among petroleum engineers, geophysicists, and geologists to determine the range of existing errors and use several variables together to estimate the amount of the desired reservoir parameters^44^. Geostatistical techniques are capable of estimating the value of the target parameter in a place where no data is available using known coordinates^45^. This feature enables GM to be considered in a wide range of exploration sciences, such as mineral resources and hydrocarbon reservoirs^43^. There is a myriad of methods in GM for interpolating the missing or needed data points, not the least of which is Kriging (Kr), employing a confined set of valuable data points to predict the value of the unavailable variable through a continuous spatial area^46^. A sharp difference between this approach and other simple interpolators, such as Gaussian decays or Distance Weighted Interpolation, is that the spatial correlation among valuable data points is utilized in the spatial field of the research area in order to interpolate target data points. However, in other methods, the weights only depend on a geometric characteristic, such as the distance. It does not change with the change in the spatial structure of the samples, and as the spatial structure weakens, the role of the samples decreases as long as the weight of all the samples is equal^47^. An added advantage of Kr is that it can provide the uncertainty surrounding each interpolated data point. Another important benefit of Kr is that the associated error for each estimate can be calculated^48^.

### Ordinary kriging (OKr)

Kriging has different methods, such as simple and Ordinary Kriging, Indicator Kriging, Universal Kriging, etc., and this study employs Ordinary Kriging (OKr) to predict the shear wave velocity. OKr is the most useful Kriging method utilizing exciting information in the neighbourhood of the target situation data points. This linear estimator method's basis is to evaluate the variable's variability structure concerning the spatial distance and local Mean. Different weights are given to the points in this method to make the data variations smoother and determine the estimation error and its validity^49^. The equation of the OKr method estimation is based on Eq. (10):

Assuming an observation vector of Z = (Z1,..,Zn) in a D-dimensional space (S1,…..,Sn), where D ∈ R^d.

We can predict the value of Z in S0, and the accidental gausi field will be derived as Z(s) = μ + δ(s) where μ is the mean and δ(.) is the error^50^. In this case, the normal kriging will be defined as a weight average as following:

10 $$\widehat{Z}\left({s}_{0}\right)={\sum }_{i=1}^{n}{\mathrm{w}}_{\mathrm{i}}\mathrm{Z}({\mathrm{s}}_{\mathrm{i}})$$

where Wi is the kriging weights. Under these circumstances the kriging will become a non-oblique prediction method.

In Eq. (10), z equals the estimated values, w_i_ equals the weight, and z_i_ equals the sample values. After estimating parameters through the OKr method, the validation of outcomes is also crucial, so following the steps mentioned above is to evaluate estimations and assess the power of the kriging method using the Cross-Validation (CVm) and Jack Knifing (JKm) methods.

### Cross-validation method (CVm)

Cross-validation is a statistical procedure that can produce roughly unbiased predictors by examining various ranges of errors, from minor to glaring, in many intricate conditions^51^. Three steps are essential for the method: First, the z_i_ data selected at random must be removed from the information one by one. Subsequently, the prediction pattern must be again estimated regarding the residual n-1 data point. The next step is checking out the re-computing way that predicts the removed data correctly; thereupon, the total estimations, as well as the removals of *z*_*i*_, must be made on average as following Eq. (11):

11 $$CV= \frac{1}{n}{\sum }_{i=1}^{n}({\mathrm{Z}}_{\mathrm{i}}-{\widehat{Z}}_{\mathrm{i}} )$$

where the ${\widehat{Z}}_{\mathrm{i}}$ is the predicted Zi value which is unavailable in the model. This type of CVm is known as leave one out cross validation (LOOCV). To calculate the error of this technique is a time-consuming task, as one needs to fit the data over n-times and calculate the (Zi-${\widehat{Z}}_{\mathrm{i}}$) prediction error until they can predict the mean. Hence, the Generalized cross validation (GCV) is a better alternative over LOOCV as it will only fit the model data once and is a more time efficient approach (Hastie et al. 2009). Additionally, the K-fold cross validation can be considered as another technique. This method removes the n/k from the model, calculates the error, and predicts the mean K (Normally valued 5 or 10). The lower the error model, the more reliable the predicted model.

The process is repeated numerously until all data subsections have been evaluated. Eventually, the average validation is presented as the last assessment^52^.

### Jack-knife method (JKm)

Turkey presented the JKM in 1958, which is a repetitive procedure that can help increase accuracy and appraise the error of the estimator. Firstly, the parameter is estimated from the whole sample^53^. Then each element is, in turn, dropped from the sample, and the desired parameter is calculated from this smaller sample^54^. If the parameter to be estimated is the population mean of x, we compute the mean ${\overline{x}}_{i}$ for each subsample consisting of all but the i-th data point (Eqs. 12–14).

The Jack-knife technique is like LOOCV (Rizzo 2019). If we remove the observation I from the observational vectors and show them as ${Z}_{\left(i\right)}=({Z}_{1}, \dots , {Z}_{i-1}, {Z}_{i+1}, {\dots , Z}_{n})$ , then, the Z(i) will equal the i observation of the jakknife value. If ${\widehat{\theta }}_{\left(i\right)}=\theta ({Z}_{1}, \dots , {Z}_{i-1}, {Z}_{i+1}, {\dots , Z}_{n}$) then the value of variance $\widehat{\theta }$ will be as following:

12 $$V\widehat{a}r\left(\widehat{\theta }\right)=\frac{n-1}{n}{\sum }_{i=1}^{n}({\widehat{\theta }}_{\left(i\right)}-{\widehat{\theta }}_{\left(.\right)} )$$

where $${\widehat{\theta }}_{\left(.\right)}= \frac{1}{n}{\sum }_{i=1}^{n}({\widehat{\theta }}_{\left(i\right)})$$

These n estimates would form a total assessment of the distribution of the sample statistic computed over many samples. In particular, the Mean of this sampling distribution is the average of these n estimates.

13 $$\overline{x} = \frac{1}{n}\sum {_{i = 1}^{n} \overline{x}_{i} }$$

A jack-knife estimate of the estimator's variance can be calculated from the variance of the distribution ${\overline{x}}_{i}$.

14 $$Var(\overline{x}) = \frac{n - 1}{n}\sum {_{i = 1}^{n} } (\overline{x}_{i} - \overline{x})^{2}$$

## Result evaluation

In this numerical research, a fair range of statistical benchmarks have been utilized to accurately assess the results and standards. Mean and standard deviation (STDEV) provide a better understanding of the estimated data and their dispersion compared to the measured value. Mean is equal to the sum of all data points divided by their numbers, and STDEV ($\sigma$) is defined in Eq. (15) as follows:

15 $$STDEV(\sigma ) = \sqrt {\frac{{\sum {_{i = 1}^{n} (x_{i} - \overline{x})^{2} } }}{n - 1}}$$

where m is the number of data points, *X*_*i*_ is each of the values of the data, and $\overline{X }$ presents the Mean of Xi. In order to evaluate the relationship between the estimated and major values, R-Squared and coefficient of determination (R^2^) and correlation (here is Corr) were implemented as Eqs. (16 and 17):

16 $$Corr = \frac{{Cov\left( {x_{i} ,x_{i(E)} } \right)}}{{\sigma x_{i} \times \sigma x_{i(E)} }}$$

17 $$R^{2} = \frac{{\sum {_{i = 1}^{n} (x_{i} - \overline{x}_{i} )(x_{i(E)} - \overline{x}_{i(E)} )} }}{{\sqrt {\sum {_{i = 1}^{n} (x_{i} - \overline{x}_{i} )^{2} \sum {_{i = 1}^{n} \left( {x_{i(E)} - \overline{x}_{i(E)} } \right)^{2} } } } }}$$

And for further investigation, these benchmarks also were used: mean absolute percentage error (MAPE), Minmax error, root mean square error (RMSE), Margin of error (ME), mean absolute error (MAE), and mean percentage error (MPE), which their equations are listed in Eqs. (18–23) as follows:

18 $$MAPE = \frac{1}{n}\sum\limits_{i = 1}^{n} {\left| {\frac{{x_{i} - x_{i(E)} }}{{x_{i} }}} \right| \times 100\% }$$

19 $$RMSE = \sqrt {\frac{1}{n}\sum {_{i = 1}^{n} \left( {x_{i} - x_{i(E)} } \right)^{2} } }$$

20 $$MAE = \frac{1}{n}\sum {_{i = 1}^{n} } \left| {x_{i} - x_{i(E)} } \right|$$

21 $$ME = \frac{{\sum\nolimits_{i = 1}^{n} {\left( {x_{i(E)} - x_{i} } \right)} }}{n}$$

22 $$MPE = \frac{1}{n}\sum\limits_{i = 1}^{n} {\frac{{\left( {x_{i} - x_{i(E)} } \right)}}{{x_{i} }}} 100\%$$

23 $$Min\max = 1 - \frac{{\sum\nolimits_{i = 1}^{n} {\frac{Min(error)}{{Max(error)}}} }}{n}$$

where x_i_ is the measured V_S_ value, x_i(E)_ is the estimated V_S_ value, $\overline{x }$_i_ is the average of the measured V_S_ value, and $\overline{x }$_i(E)_ the figure for the estimated value, on average, and n is the number of samples. Furthermore, $\sigma$ is the standard deviation, and Cov is the covariance, respectively^2,55^.

## Results and discussion

### Estimation by ERR

**Figure 2:**
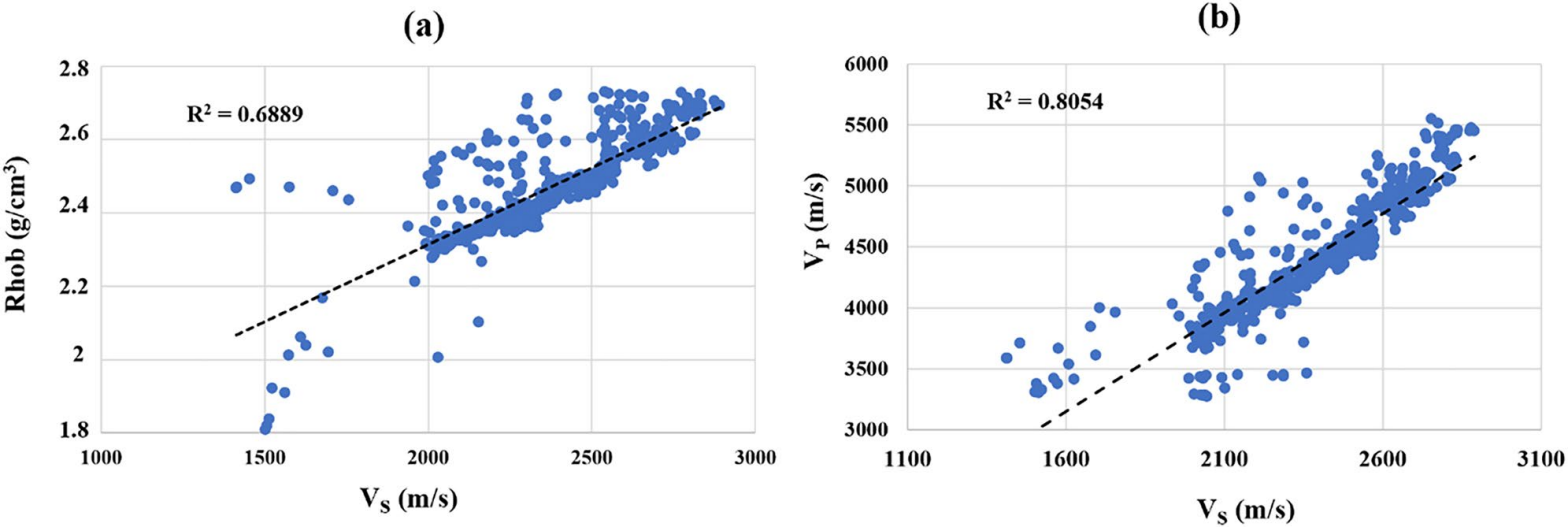
The correlation between (**a**) Rhob with R^2^ = 0.6902 and (**b**) V_P_ with R^2^ = 0.8054 versus V_S_ in the target well.

This research to estimate V_S_ through ERR uses P-wave velocity and density, whose correlations with shear wave velocity are examined in the first step. In Fig. 2a and b, it was found that their Correlation were R^2^ = 0.68 and R^2^ = 0.80 for density and V_P_ with V_S_, respectively. Therefore, it is expected that the result of the estimation V_S_ using V_P_ would be more satisfying than when density is used. Table 2 contains information on the Mean and STDEV of the original (measured), which are 2407.29 and 260.52, respectively.

**Table 2:** Statistical information of origin S–wave velocity.

Parameter	Mean	STDEV
S-wave velocity	2407.29	260.52

Regarding the obtained results using ERR in Tables 3 and 4, among rock physics relationships, the G.C method is responsible for the best performance in estimating V_S_. This method could obtain R^2^ = 0.8104 with a correlation of 0.9002. There is a significant difference between the values of MPE, ME, and MAE in the G.C method and those in other rock physics approaches as they are almost 3, 7, and 70, respectively, for the G.C method. Moreover, the G.C methods accounted for the least RMSE and Minmax error, with 116.908 and 0.5254, respectively. Comparing Tables 2, 3 and 4 reveals that the mentioned method has the lowest Mean (7.83) and STDEV (1.15) difference compared to the original values of V_S_. Table 4 indicates that Castagna et al. (1993) approximately put on a similar performance with the G.C method, and this point should be mentioned that the obtained results are better when limestone line is input. To be more accurate, the figures for R^2^ for the limestone and dolomite lines equations are 0.8117 and 0.8181, and those for correlation are 0.9039 and 0.9016, respectively.

**Table 3:** Result for empirical rock physic relationship to estimate S-wave velocity (m/s).

Method	Mean	STDEV	R^2^	Corr	MAPE	ME	MAE	MPE	RMSE	Minmax
Gardner^36^	2577.33	**507.28**	**0.6766**	**0.8225**	11.129	170.129	265.212	0.0643	370.092	0.8067
Castagna et al.^16^	2672.05	403.53	0.8054	0.8974	11.375	258.655	278.523	0.1044	328.204	0.60798
Kerif (1990)	2687.85	353.09	0.8061	0.8978	12.183	280.508	293.468	0.1158	324.3661	0.5952
Greenberg and Castagna^18^ (G.C)	**2415.12**	**261.67**	**0.8104**	**0.9002**	**3.1668**	**7.8843**	**70.322**	**0.0050**	**116.908**	**0.5254**

Significant values are in bold.

**Table 4:** Result for empirical rock physic relationship to estimate S-wave velocity using Castagna et al. (1993).

Method	Mean	STDEV	R^2^	Corr	MAPE	ME	MAE	MPE	RMSE	Minmax
Limestone. Line	2396.69	248.55	0.8171	0.9039	3.14	10.33	69.725	0.0020	112.63	0.5156
Dolomite. Line	2523.67	273.05	0.813	0.9016	5.5955	116.42	128.746	0.0503	166.42	0.5529

Furthermore, the values of RMSE and MAE for the dolomite line are much higher than those for the limestone line, but no significant difference was seen in other benchmarks. The differences between the estimated and measured (original) mean shear wave velocities for the limestone and dolomite lines methods are 10.6 and 116.38, and the difference in STDEV is 11.97 and 12.53, respectively. Since these methods, in addition to P–wave velocity, employed lithological information; therefore, the obtained results are much more accurate and satisfactory. Castagna et al. (1985) and Kerief's (1990) equations work only for P–wave velocity and evaluating the results through benchmarks shows that there are no sharp differences between the performances of these methods, so the figures for R^2^ and Correlation for them are approximately 0.80 and 0.89 (Table 3). The STDEV for Castagna et al. (1985) is 403.53, whereas that for Kerif is 353.09, but the Mean for both methods is almost the same. As expected, Gardner's equation (1974) reveals the weakest performance among all used ERR here. Table 3 shows this equation stands at least in R-squared and Correlation, 0.6766 and 0.8225, respectively, although it is responsible for the highest amounts of Minmax (0.8067) and RMSE (370.092).

**Figure 3:**
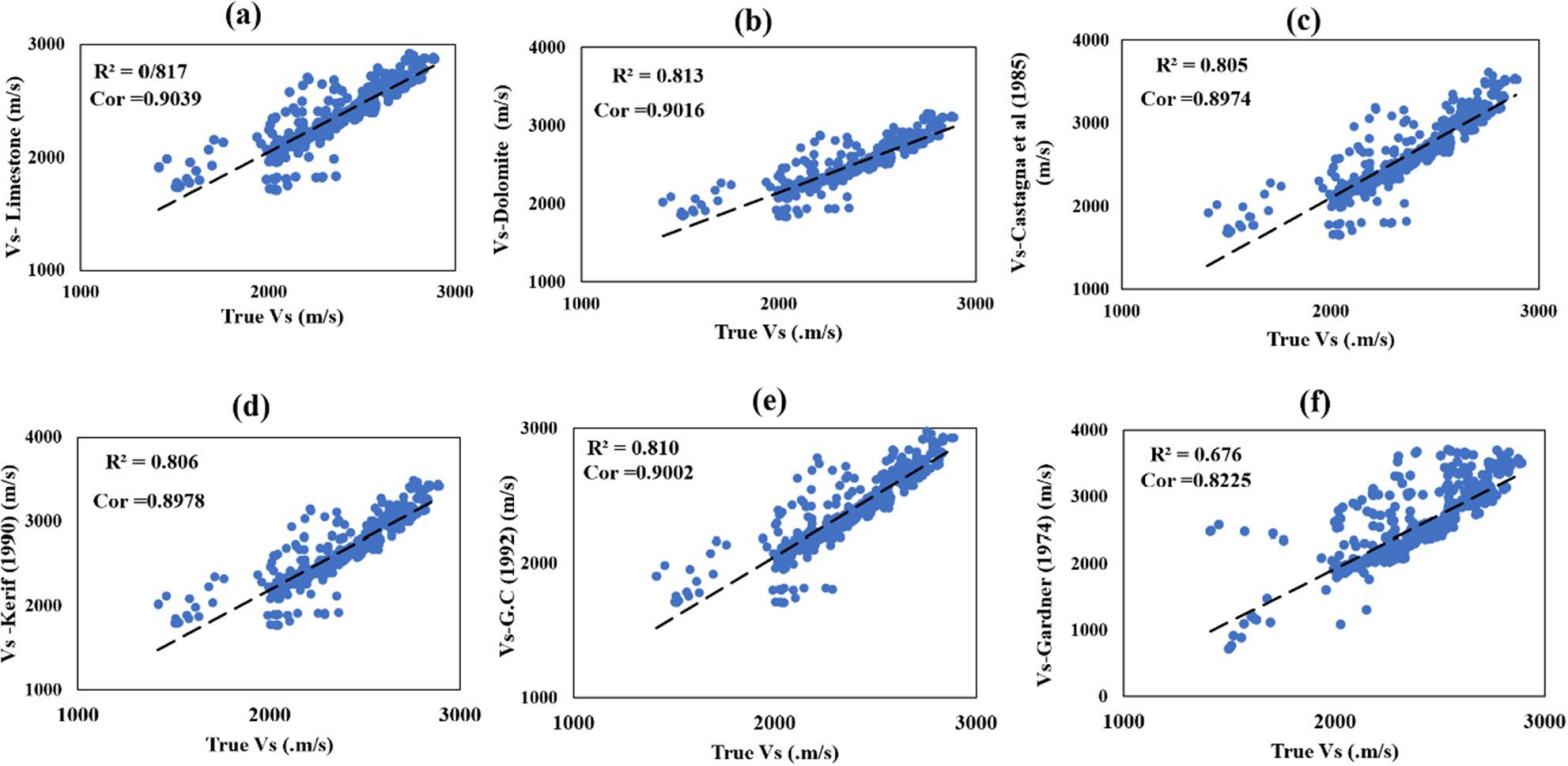
The correlation between (**a**) V_S_. Limestone with R^2^ = 0.817 = 0.8054, (**b**) V_S_. dolomite. Line with R^2^ = 0.813, (**c**) Castagna et al. (1985) with R^2^ = 0.805, (**d**) V_S_. Kerif (1990) with R^2^ = 0.806 (**e**) V_S_. Greenberg–Castagna (1992) with R^2^ = 0.810, and (**f**) V_S_. Gardner (1974) with R^2^ = 0.676 against Vs.

**Figure 4:**
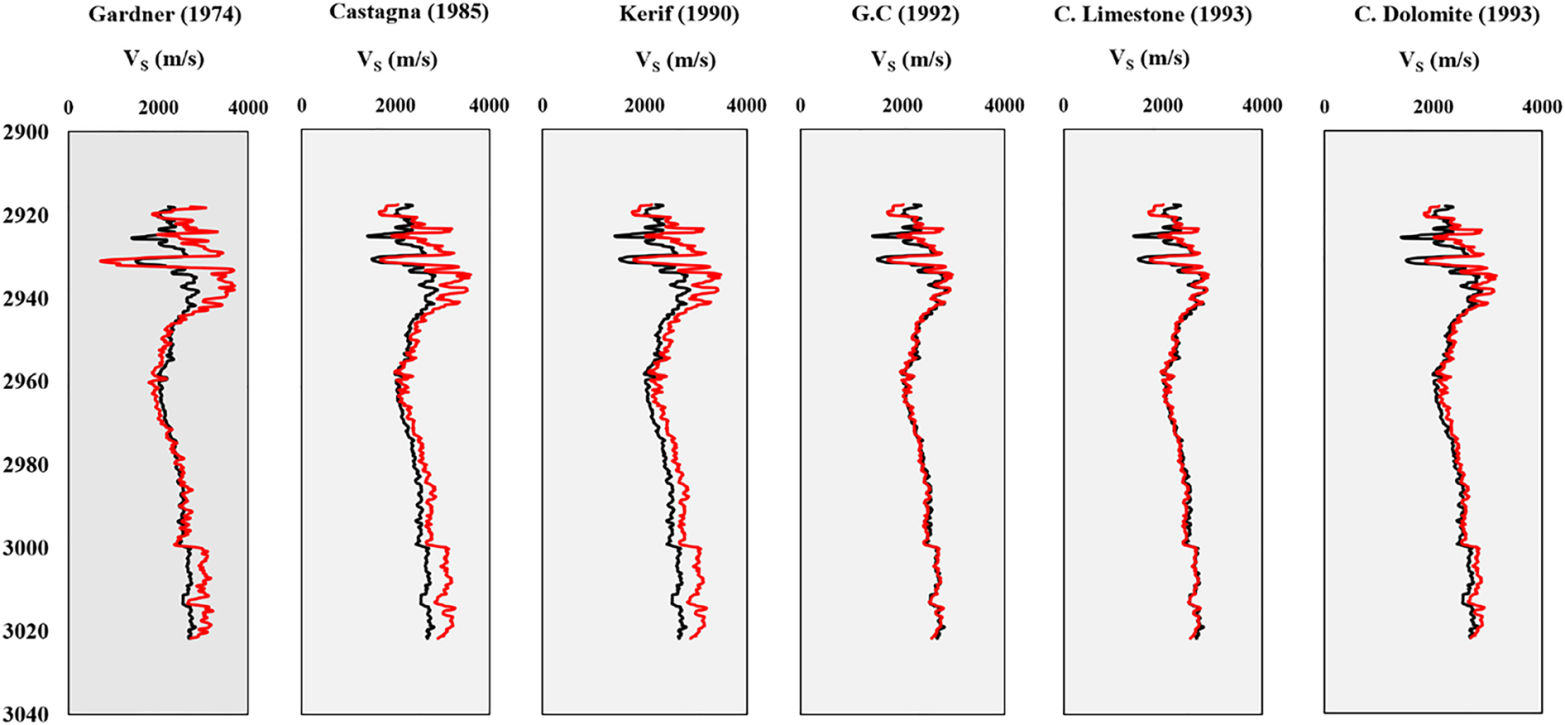
Well-logging plots for estimated shear wave velocity by ERR (red curves) and original shear wave velocity (black curves) in well A.

Additionally, it has the largest difference in standard deviation compared to the original shear wave, with 246.76. In other statistical benchmarks, the errors are high and similar to Castagna et al. (1985) and Kerif (1990), not G.C and Castagna et al. (1993). So at the end of this part, it was clear that the poorest results belong to Gardner's equation (1974), and they are to be improved by GM in the next sections. Figure 3 shows all cross plots for ERR for estimating VS in well A, and Fig. 4 illustrates well–logging plots for ERR results.

### Estimation by GM

As stated before, the shear wave velocity estimated by Gardner^36^ was the poorest result, and in this section, GM strives to improve it. Hydrocarbon reservoirs usually involve various stratigraphic layers separated by surfaces referring to geological processes such as deposits over time. The layers, more often than not, have a different thickness (proportional), or maybe some of their parts are unavailable due to being missing as a result of tectonic events such as faults or experiencing erosion (truncation). Sometimes, onlap occurs, in which layers follow the available top without erosion; however, they complete the topography; therefore, the stratigraphy grid must be corrected. And finally, strata are not matched with the upper and lower layers (combination). Given those considerations, in the first step to employ GM, to regularize and better understand the spatial relationships of variables, layers for the study area must be converted to a regular form.

24 $$Z_{Cor} = \frac{{Z{}_{t} - Z_{bottom} }}{{Z_{top} - Z_{bottom} }} \times T_{av}$$

where Z_bottom_ and Z_top_ are Correlation at the base and top, Zt is for the target layer, Tav is responsible for the study area's average thickness, and Z_Cor_ illustrates Z coordinator^56^.

After generating a coordinate system, estimations of shear wave velocity in the target well are followed in two directions:

- Using the available density and through CVm and JKm.
- Using the density of two nearby wells, including wells B and C employing JKm.

**Figure 5:**
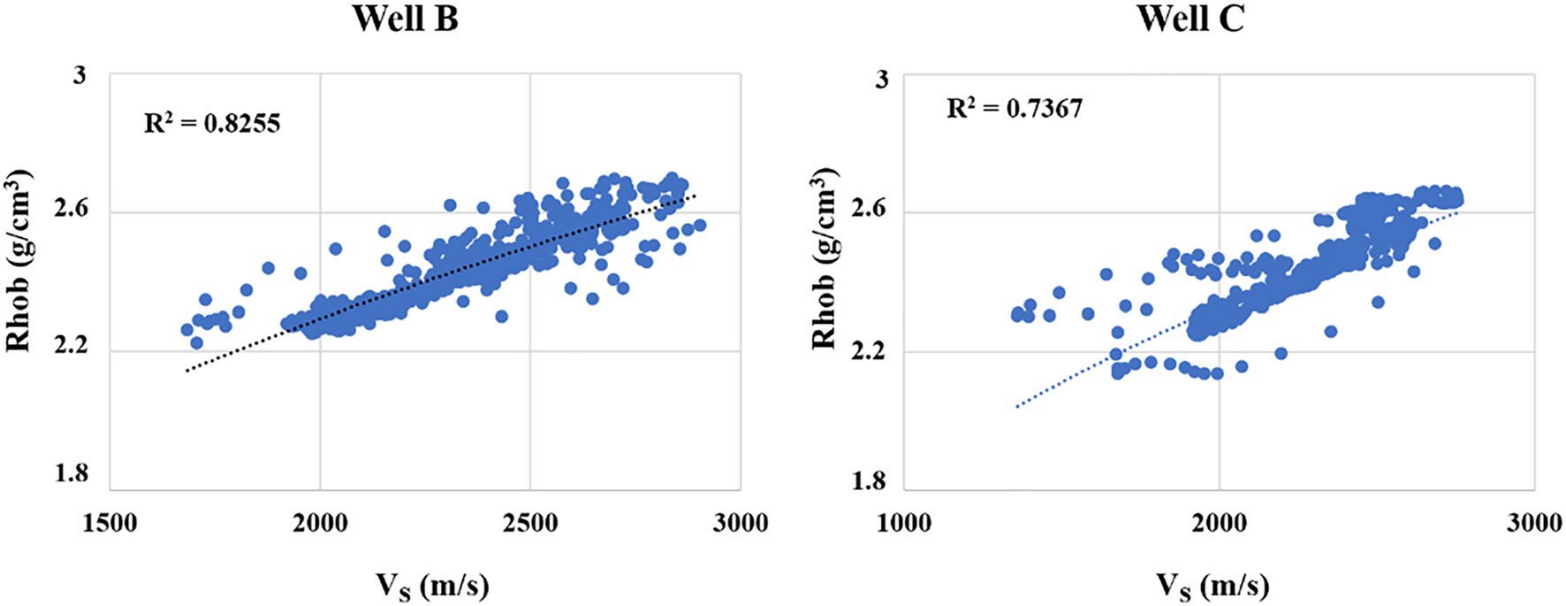
The relationship between shear wave velocity and density for wells B and C.

The relationship between shear wave velocity and density must be investigated in two directions. In the target well, it was assessed and shown in Fig. 2 with R^2^ = 0.6889, and Fig. 5 illustrates the correlations for wells B and C with R^2^ = 0.8255 and R^2^ = 0.7367, respectively.

It is clear that in wells B and C, there is a stronger relationship between S–wave velocity and density compared to the target well, and well B accounted for the best relationship (R^2^ = 0.8255). Next, all coefficients for Garder's equation must be localized, and their results are listed in Table 5.

**Table 5:** Gardner (1974) localized coefficients for the used wells.

Well	a	b
Well A	0.12	0.38
Well B	0.11	0.36
Well C	0.17	0.34

**Figure 6:**
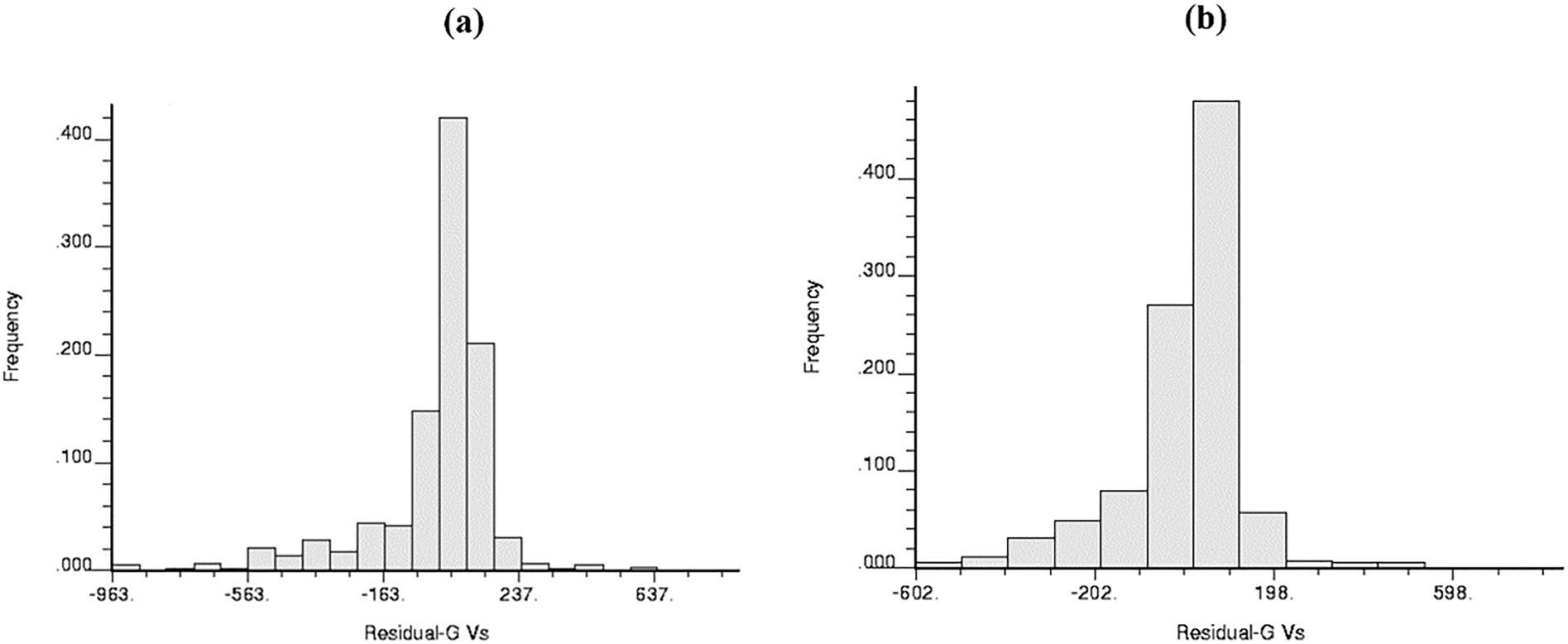
(**a**) Frequency plot of Residual S- wave for Gardner (1974) (**a**) using one well (**b**) using nearby wells.

**Figure 7:**
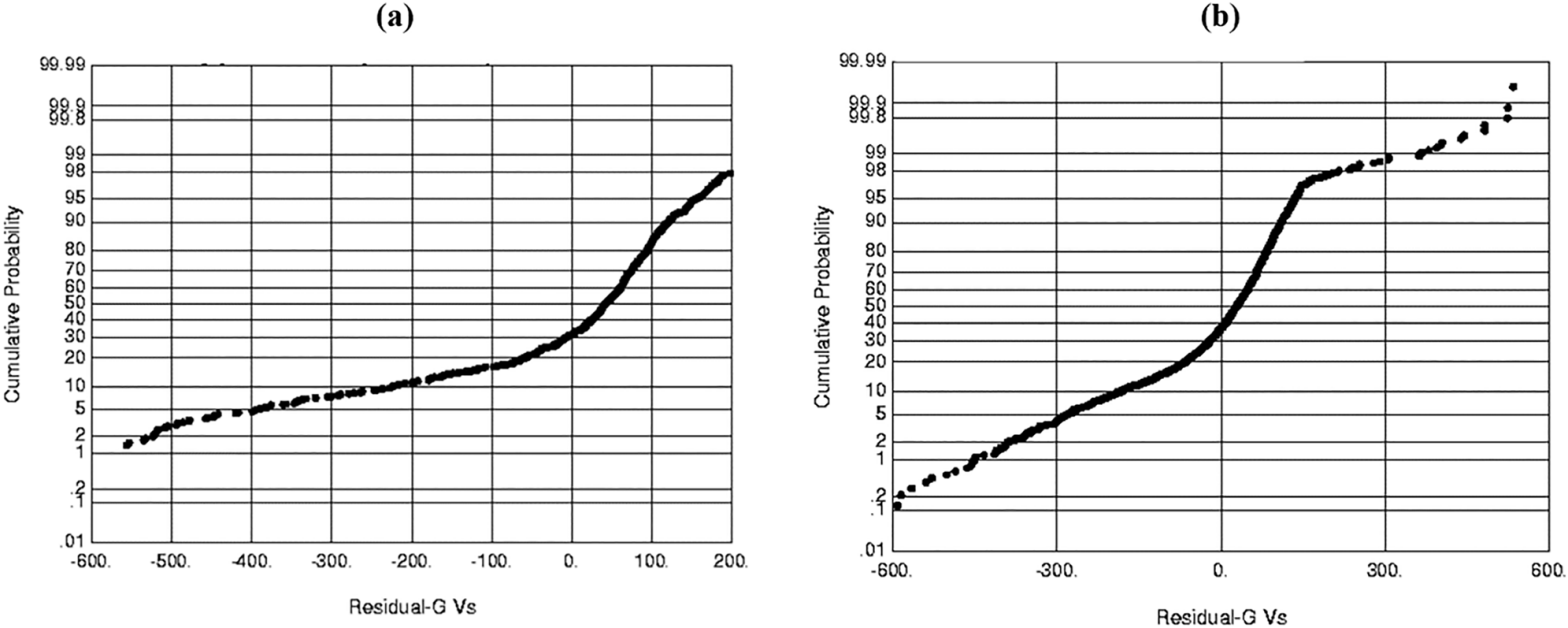
(**a**) Probability plot of Residual S- wave for Gardner (1974) (**a**) using one well and (**b**) using nearby wells.

Shear wave velocity was estimated through Gardner's equation (1974) using the local coefficients that its absolute value of the difference with the measured V_S_ would equal residual velocity (RV); from now on, all geostatistical processes will be done on RV. Data preparation is one of the most important steps in GM, not the least of which is normalizing the used data-set. This is because it allows various data from different parts of the study to be compared, and normalization puts the data in a similar domain by removing or reducing irrelevant data or data anomalies. Additionally, since the Kriging used in this research is linear, all data must be normalized. Therefore, during the following step, the normal distribution of RV should be examined for the two directions; this point should be mentioned that for JKm in the second direction, two calculated RV are placed one after the other (considered as an RV for two nearby wells). For this purpose, this paper uses two graphical approaches: frequency histograms and probability plots. Figure 6 shows that the frequency histograms of two directions are almost bell-shaped, and the probability plots are close to the straight line in Fig. 7. It can be concluded that their distributions are normal and ready to be used for geostatistical estimates. Following this, the spatial relationship between the data must be investigated through variography, known as the first and most important step in GM after data preparation. This essential step is implemented through a variogram. This powerful and invaluable tool can supply an array of information on the spatial explication of the data by appraising the proportion of change compared to distance^57^; therefore, in the stage after, the empirical variogram has been generated on RV to provide the intensity of spatial changes of variables (a) using one well and (b) using nearby wells.

**Figure 8:**
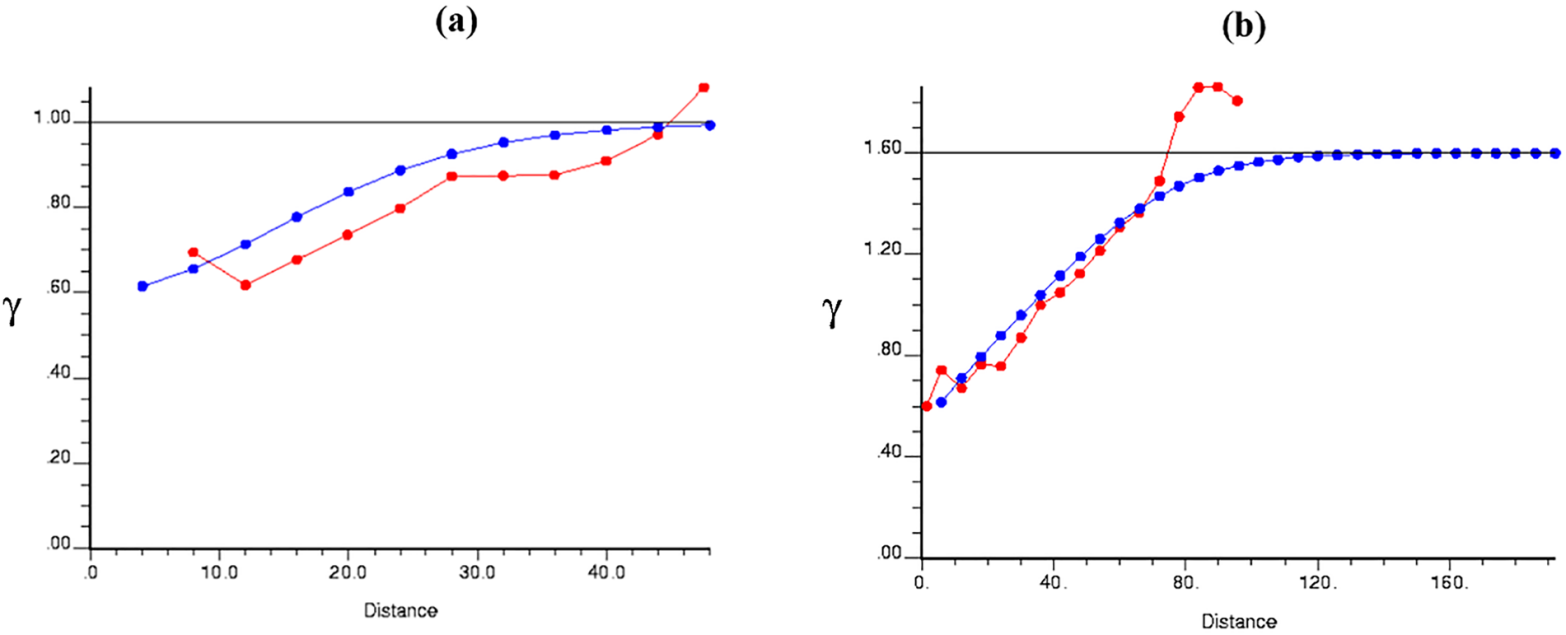
The empirical variogram (red diagram) and the model fitted to it (blue diagram) for the density for (**a**) using one well (**b**) using nearby wells.

A standard variogram model was fitted after estimating the empirical variogram to accurately investigate the data's special structure (Fig. 8). This is because using models results in variography being done in all directions and distances, and the errors due to lack of information and reaching real values would be minimized^58^. The survey uses the Gaussian model when the accessible density (from the target well) is used and the exponential and Gaussian models when the information from two nearby wells is used (Fig. 8). In the following stage, S-wave velocity was estimated through OKr, and then to assess the productivity and performance of OKr, CVm and JKm were employed. CVm relies on data that has been observed but not used when building the model. These data are used to check and measure the model's efficiency to predict new data. To apply CVm, an abandon-one-out strategy was implemented on the target well's data set (on the residual velocity). In JKm, when the information of one well was put into effect, six points at seven-point distances from the studied zone (the target well) were ignored, after which the shear wave speed was estimated through the remaining points from the data set. And when information from two nearby wells was involved, the whole V_S_ of the target well was ignored, so it has been estimated by employing the data sets of wells B and C by JKm.

Comparing Tables 3 and 6 reveals that the GM method could effectively improve Gardner's results equation for shear wave velocity estimation, as R^2^ changed from 0.6766 to 0.8865 and 0.8082 for CVm and JKm, respectively. And there is a positive change in the correlation of about 8% for JKm and 12% for CVm. The Mean difference and STDEV results obtained by GM with measured shear wave velocity are much less than the Gardner method's results, and the difference for Mean in both GM is under three units, and that of for STDEV in CVm and JKm are 9.02 and 53.47. Amongst all statistical benchmarks, ME and RMSE show more drastic change, especially in CVm, as the former decreased from 170.129 to under two units, and the latter registered a significant fall of approximately 280 and 230 units in CVm and JKm, respectively. The other benchmarks' figures include MPAE, MAE, and Minmax reduced in both used GM. Moreover, given those considerations, it can be deduced that CVm achieved much better results in predicting S–wave velocity by Gardner's equation (1974) than JKm.

**Table 6:** The S-wave velocity estimation results using the CVm and JKm for Gardner (1974) method.

Method	Mean	STDEV	R^2^	Correlation	MAPE	ME	MAE	MPE	RMSE	Minmax
CVm	2408.24	269.53	0.8865	0.9415	2.3434	0.95472	53.3275	0.0089	91.330	0.5586
JKm	2405.79	313.99	0.8082	0.899	3.4605	1.5291	75.1255	0.0016	138.7626	0.7170

**Figure 9:**
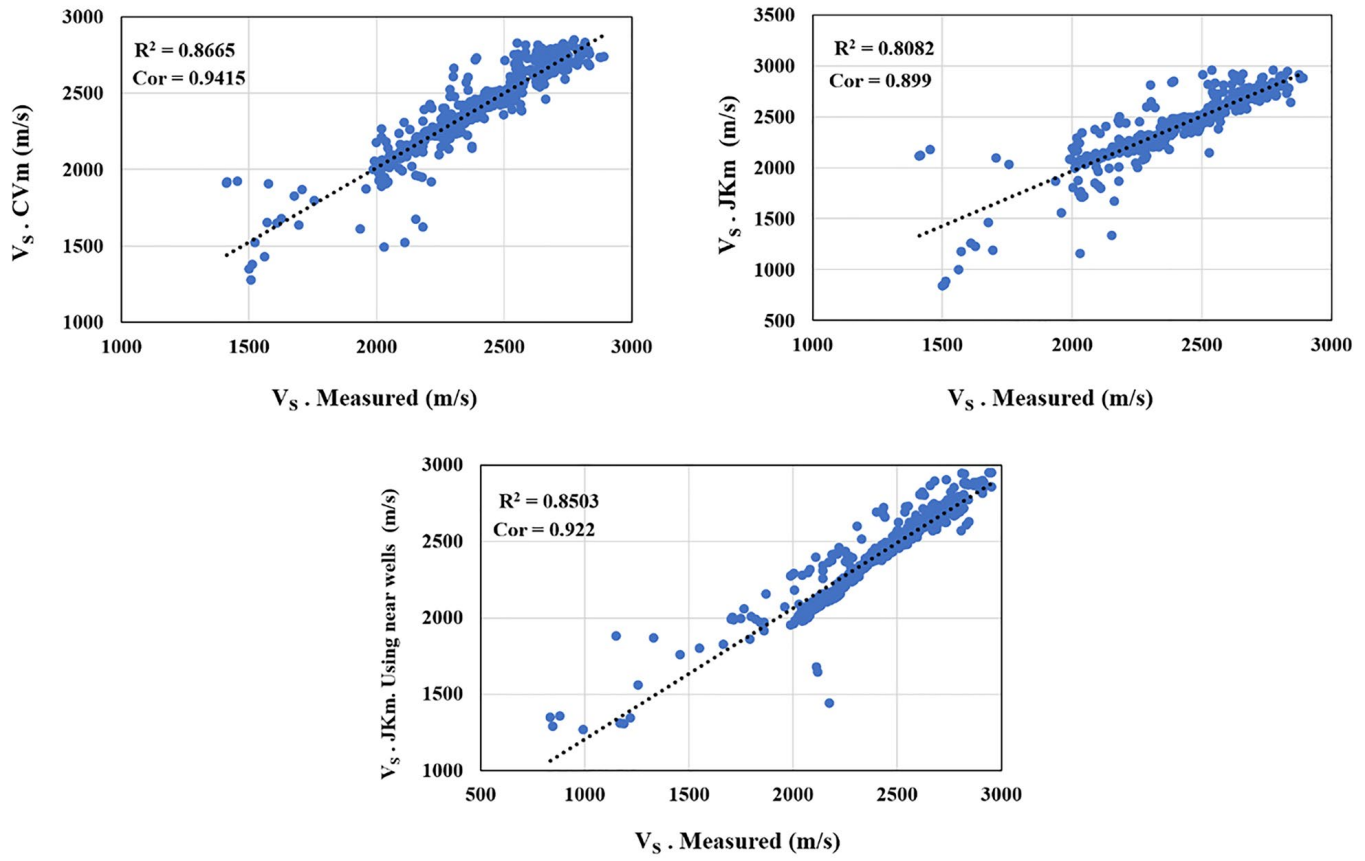
The measured V_S_- estimated cross plots utilizing CVm and JKm in the study area.

**Figure 10:**
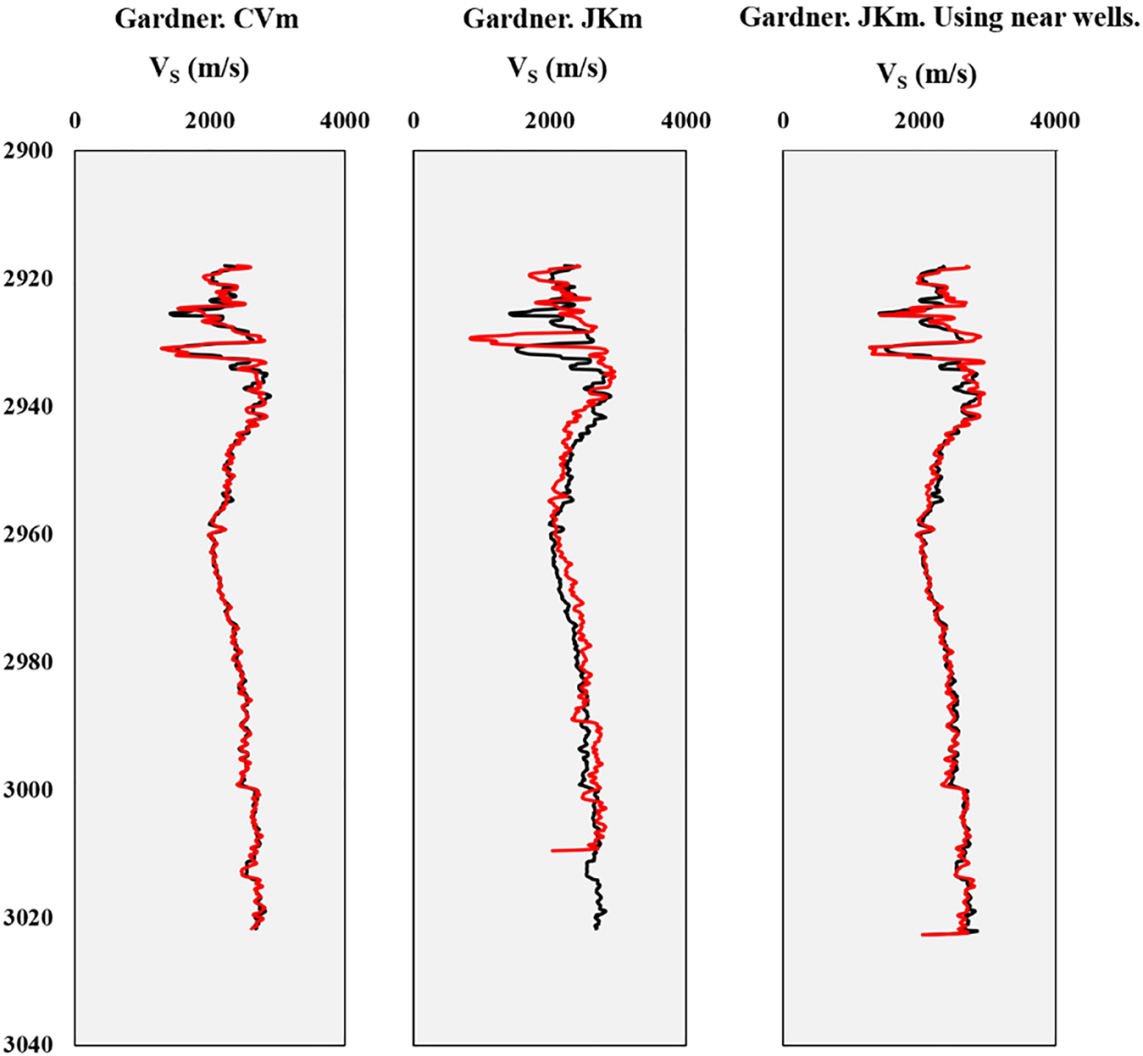
Well-logging curves for shear wave velocity estimation using CVm and JKm (red curves) and measured shear wave velocity (black curves) in in the study area.

Based on the findings presented in Table 6, the novel approach implemented through JKm demonstrates remarkable success in predicting S-wave velocity. The analysis reveals a high level of prediction accuracy, with an R2 value of 0.8503, a correlation coefficient of 0.922, and an RMSE value of 109.5701. Notably, this approach significantly reduces the differences between the mean and standard deviation of the measured shear wave velocity, with approximate reductions of 41 and 31, respectively, compared to Gardner's results (Table 3). Furthermore, Table 7 showcases the effectiveness of the new strategy in improving Eq. 6. Using the Rhob-VS relationship from nearby wells, as applied by JKm, is highly successful and can yield excellent results. The improvements achieved through this approach validate its applicability and effectiveness in estimating S-wave velocity in the target well. In conjunction with these quantitative results, Figs. 9 and 10 provide visual representations of the relationship between measured VS and estimated Vs and depth profiles of the estimated Vs compared to the measured Vs in the study area. These graphical representations further support the efficacy of the JKm approach in accurately estimating S-wave velocity.

**Table 7:** The S-wave velocity estimation results using JKm for Gardner's method (1974).

Method	Mean	STDEV	R^2^	Correlation	MAPE	ME	MAE	MPE	RMSE	Minmax
JKm	2446.527	283.051	0.8503	0.922	3.1192	3.5092	72.041	0.00142	109.5701	0.5705

Considering the obtained results, ERR can estimate shear wave velocity in wells, which is not measured very well using P-wave velocity, and the results would be improved if lithology information was used in addition to VP. However, a myriad of studies using P-wave velocity, lithology, and petrophysical information have tried to estimate S-wave velocity through intelligence methods, not the least of which are machine and deep learning techniques. Some of them are listed as follows: Li et al. (2017), Hu et al. (2020), You et al. (2021), Luo et al. (2022)^59–62^**.** (It must be noticed that other similar studies were mentioned in the pervious sections such as Anemangely et al.^32^, Wang and Cao^25^, Wang et al.^13^, Zhang et al.^2^**)**. In these studies, at least six or seven sets of input data (well-logging data) have been put into effect to predict accurately, which is not economical or time-efficient. Still, here it was tried to improve an ERR successfully using only the parameter density to estimate S-wave velocity, and a very acceptable result was obtained Corr = 0.94, which means there is a difference of just one or two percent in compression because that research utilized more than six or seven inputs.

On the other hand, most studies were conducted to predict V_S_ only for well a and did not pay attention to estimating S–wave velocity using other wells, and very few studies that have used multiple wells for estimation have used a large number of their data for estimation in the target well^12^. The present research takes a useful and further step and could estimate V_S_ by employing information from other wells without using the information of the target well.

Given these considerations, the results reveal that the performance of OKr, CVm, and JKm in estimating shear wave velocity is more efficient than earlier research, as they were capable of predicting VS with far less data points yet with perfect accuracy.

It must be mentioned that the new strategy in this research can be effectively employed in other geological content with similar properties such as porosity, density, and fluid content. However, it may not work in high-porosity porous media, fractured zones, or zones containing remarkable amounts of shale. Because in the mentioned porous media, the density changes are remarkable, and more advanced numerical methods are needed. Also, we think the suggested numerical methods in gas zones may not function effectively. These are important issues that we plan to address in future research.

## Conclusions

This research generated a novel numerical direction for shear wave velocity estimation based on geostatistical methods through three carbonate wells next to each other. In the first section, among ERR methods, those involving lithology and P–wave velocity in the estimations obtained better results. The Greenberg–Castagna (1992) equation accounted for the best result with Corr = 0.90, while Gardner's (1974) equation displayed the poorest results with R^2^ = 0.67 and Corr = 0.82. This research employed GM to improve the results through the OKr approach, CVm, and JKm to assess the results. Accordingly, the results improved from Corr = 0.82 to Corr = 0.94 and Corr = 0.89 when CVm and JKm were employed, respectively. A detailed analysis of results using ten statistical benchmarks shows that the two methods (CVm and JKm) could improve results effectively, but CVm provided better performance than JKm when available information about only the target well was used.

Additionally, JKm could effectively estimate (R^2^ = 0.85, Corr = 0.922) the target well Vs using a data set of two nearby wells without any information about the target well. As a whole, this case study research shows that GM has considerable potential to improve shear wave velocity estimation by density, while there is no good correlation between them in compression with the V_P_–V_S_ relationship, which has an excellent correlation. The next studies can implement the recommended numerical directions in other fields with different lithologies, such as sandstone, highly-shale, or fracture reservoirs.

## Data Availability

The datasets used and/or analyzed during the current study are available from the corresponding author on reasonable request.
